# Comparison of *Polygonatum sibiricum* Polysaccharides from Different Extraction Methods

**DOI:** 10.3390/foods14132188

**Published:** 2025-06-23

**Authors:** Yan Chen, Hanchen Du, Wenjie Qu, Chaoqun Sun, Qu Chen, Yuping Du, Zhuoyuan Zhang, Yiran Guo, Chonglin Wang, Jian Huang, Jiyong Yin

**Affiliations:** Key Laboratory of Public Nutrition and Health, National Health Commission of the People’s Republic of China, National Institute for Nutrition and Health, Chinese Center for Disease Control and Prevention, Beijing 100050, China; chenyan7341@163.com (Y.C.); duhc@ninh.chinacdc.cn (H.D.); qwj370612@163.com (W.Q.); sunchaoqun97@163.com (C.S.); chenqu@ninh.chinacdc.cn (Q.C.); duyp@ninh.chinacdc.cn (Y.D.); zhangzy@ninh.chinacdc.cn (Z.Z.); guoyr@ninh.chinacdc.cn (Y.G.); wangcl@ninh.chinacdc.cn (C.W.); huangjian@ninh.chinacdc.cn (J.H.)

**Keywords:** *Polygonatum sibiricum* polysaccharide, extraction method, ultrasound-assisted extraction–deep eutectic solvents (UAE–DESs), structural composition, antioxidant activity

## Abstract

Although our previous research has indicated that the ultrasound-assisted extraction–deep eutectic solvent method possessed the highest extraction yield for *Polygonatum sibiricum* Polysaccharide, it is uncertain whether the *Polygonatum sibiricum* Polysaccharide that was extracted by the ultrasound-assisted extraction–deep eutectic solvent method possesses the same performance as that extracted by other methods and whether separation and purification affect the performance of *Polygonatum sibiricum* Polysaccharide. This paper aimed to compare the differences in performance among the *Polygonatum sibiricum* Polysaccharides extracted using different methods, including the ultrasound-assisted extraction–deep eutectic solvent method, which was the first method used by our team to extract *Polygonatum sibiricum* Polysaccharide. This paper also aimed to compare the differences in *Polygonatum sibiricum* Polysaccharides before and after they were separated and purified. The extraction yield, structural composition, and antioxidant activity of *Polygonatum sibiricum* Polysaccharides were compared, respectively. The extraction yield (45.08%) obtained by the ultrasound-assisted extraction–deep eutectic solvent method was the highest (*p* < 0.05), the structural compositions of *Polygonatum sibiricum* Polysaccharides extracted using different methods were similar, and the separated and purified *Polygonatum sibiricum* Polysaccharide was a neutral polysaccharide. The 2,2–diphenyl–1–picrylhydrazyl and 2,20–azino–bis (3–ethylbenzothiazoline–6–sulfoniacid) levels in the *Polygonatum sibiricum* Polysaccharide extracted by the ultrasound-assisted extraction–deep eutectic solvent method were significantly higher than those obtained using other methods (*p* < 0.05), and the antioxidant activity of the *Polygonatum sibiricum* Polysaccharide extracted by this method was significantly higher after it was separated and purified (*p* < 0.05). This study not only proved that the ultrasound-assisted extraction–deep eutectic solvent method could increase the extraction yield and keep the structural composition and antioxidant activity of *Polygonatum sibiricum* Polysaccharide at the maximum levels but also confirmed that the extracted *Polygonatum sibiricum* Polysaccharide should not be further separated and purified, providing a potential technique to extract *Polygonatum sibiricum* Polysaccharide. This study will further promote the application of *Polygonatum sibiricum* Polysaccharides in the health product industry.

## 1. Introduction

*Polygonatum sibiricum* (*P. sibiricum*) belongs to the Liliaceae family and the Polygonatum genus and is a traditional medicinal material in China [1]. There are three source plants in the Pharmacopeia, which include *Polygonatum sibiricum Red (P. sibiricum Red)*, *Polygonatum cyrtonema Hua (P. cyrtonema Hua)*, and *Polygonatum kingianum Coll. et Hemsl (P. kingianum Coll. et Hemsl)* [2]. The functional components of *P. sibiricum* mainly include polysaccharides, saponins, flavonoids, total phenols, etc., and its biological activity depends on its chemical composition [3]. *P. sibiricum* can enhance the immune system and strengthen the function of the spleen [4]. As a medicinal and edible plant, *P. sibiricum* has received more and more attention due to its distinguished pharmacological activity and edible value.

*Polygonatum sibiricum* Polysaccharide (PsP), which is the main active component of *P. sibiricum,* is abundant in roots [5]. Modern pharmacological studies have proven that PsP has various functions, such as reducing blood glucose; antioxidant, anti-tumor, and immune-regulating effects; and potential benefits in enhancing memory and preventing and treating dementia [6]. In addition, PsP’s pharmacological effects also involve stimulating cell maturation, differentiation, and proliferation, and improving the balance of host cells, thus enhancing the responsiveness of host cells to hormones and other physiological factors [7,8].

Many methods can extract PsP, and different methods might lead to different effects on the biological activity and application of PsP. The water–ethanol extraction (WEE) method is a conventional extraction method that extracts PsP through a polar solvent according to the principle of “like dissolves like” [9]. It is the most common extraction technique, and its advantages include being simple, safe, and cost-effective and the ability to easily remove interfering substances; however, its extraction yield is poor, so it is difficult to use this method to meet the practical requirements in the food production industry [10,11]. The ultrasonic-assisted extraction (UAE) method uses ultrasonic waves to accelerate the movement speed of polysaccharide molecules and improve the penetration effect of the solvent so that polysaccharides can be quickly dissolved in the solvent [12,13,14]. This method has various advantages, including a lower extraction temperature, shorter extraction time, less solvent consumption, and a smaller destructive effect on the structure of the polysaccharide. Lin et al. [15] determined the optimal conditions of the UAE method in extracting PsP, with an extraction yield of 10.86% under optimal conditions (the solid–liquid ratio was 1:20 at 810 W for 20 min). Jing et al. [16] compared the extraction yield of the WEE and UAE methods for PsP, with the results indicating that the UAE method had a higher extraction yield and was more suitable for extracting PsP than the WEE method. The deep eutectic solvent (DES) method applies a new type of “green solvents”, which consists of a hydrogen bond donor and a hydrogen bond receptor, to extract PsP. This method is safe, efficient, and energy-saving [17]. Tang et al. [18] studied the in vitro biological activity of PsP between the DES and WEE methods, and their results showed that both the extraction yield and antioxidant activity of the DES method were higher than the WEE method. The ultrasound-assisted extraction–deep eutectic solvent (UAE–DES) method is a combination of the UAE and DES methods, and it integrates the advantages of both. We have completed the optimization of the conditions for the UAE–DES method to extract PsP in our previous studies, in which the extraction yield could be higher than 40.00% under a solid–liquid ratio of 1:26 (g–mL), extraction temperature of 80 °C, ultrasonic time of 51 min, and ultrasonic power of 82 W [19].

There are many separation and purification methods for PsP, such as the membrane separation method, fractional precipitation method, and chromatographic column method [20]. The membrane separation method utilizes the selective permeability of the semi-permeable membrane to separate polysaccharide according to its characteristics in size, shape, and charge. The fractional precipitation method utilizes the different solubility of polysaccharide in different solvents (such as ethanol and acetone) to complete separation for different components of polysaccharide. The chromatographic column method, the most widely used technique, includes ion exchange chromatography and gel filtration chromatography [21]. The anion exchange chromatography, which is one kind of ion exchange chromatography, can exchange with the negative charge of a sample and is often used in the first step of separating and purifying PsP. Then, the neutral component and acidic component of PsP are obtained [22]. After that, gel filtration chromatography further separates PsP into different components according to molecular weights [23]. Finally, separation and purification are completed.

Although we have determined that the UAE–DES method possessed the highest extraction yield and a certain antioxidant activity, a systematic comparison between the UAE–DES method and other methods remains lacking, and the comprehensive effect of the UAE–DES method on PsP was not proved in our previous study. Therefore, it is still uncertain whether the UAE–DES method is the optimal extraction method for PsP. According to the above analysis, it is greatly significant to evaluate the comprehensive effects of the above four different methods, which can contribute to confirming whether the UAE–DES method is useful for extracting PsP. The UAE–DES method was proposed on the basis of the WEE method, UAE method, and DES method. Therefore, we compared the WEE method, UAE method, and DES method with the UAE–DES method in this study, so as to ensure comparability. In the future, we will further conduct more comparisons between the UAE–DES method and other methods, including microwave extraction, supercritical CO_2_ extraction, etc., so as to prove the advantages of the UAE–DES method to a greater extent. In addition, there is no report about the difference in biological activity between extracted PsP using the UAE–DES method and its main component after separation and purification. So, it is also necessary to confirm whether the separated and purified PsP can possess better functional activity through its special structures.

This study aimed to compare the performance of extracting PsP among WEE, UAE, DES, and UAE–DES methods, and to compare the performance of the extracted PsP using the UAE–DES method and its main component after it is separated and purified. The comparison included extraction yield, structural composition, and antioxidant activity. We hope this research can prove that the UAE–DES method can not only increase efficiently the extraction yield for PsP, but also keep PsP’s biological activity, so as to further promote PsP’s utilization in the health product industry.

## 2. Materials and Methods

### 2.1. Materials

*P. sibiricum* comes from Beijing, China. The chemicals used were as follows: The 2,2–diphenyl–1–picrylhydrazyl (DPPH) was purchased from Shifeng biological technology Co., Ltd. (Shanghai, China). The 2,20–azino–bis(3–ethylbenzothiazoline–6–sulfonic acid) (ABTS), Na_3_PO_4_, and H_8_M_O_N_2_O_4_ were purchased from Aladdin Co., Ltd. (Shanghai, China). The bovine serum albumin (BSA) was purchased from Sigma Co., Ltd. (St. Louis, MO, USA). The D–Glucose anhydrous–RM was purchased from Manhagebio-tech, Co., Ltd. (Beijing, China). The BCA protein assay kit and total antioxidant capacity assay kit with the FRAP method were purchased from Beyotime Biotechnology Co., Ltd. (Shanghai, China). The catalase (CAT) assay kit was purchased from Solarbio life science, Inc. (Beijing, China). The TSK–gel G3000 PWXL column was purchased from Lubex Scientific Instrument, Co., Ltd. (Guangzhou, China). The DEAE–Sepharose Fast Flow and Sephadex G–50 column were purchased from Yuanye Biotechnology Co., Ltd. (Shanghai, China).

### 2.2. Main Instruments and Equipment

The main instruments and equipment included an Allegra x–22 R centrifuge (Beckman coulter, Inc., Brea, CA, USA); SpectraMax I3X Enzyme marker (Molecular Devices Instruments Ltd., San Jose, CA, USA); digital ultrasonic cleaner (Kunshan Ultrasonic Instruments Co., Ltd., Kunshan, China); vortex mixing device ORTEx Genius (IKA, Inc., Staufen, Germany); magnetic stirrers (IKA, Inc., Staufen, Germany); U–3900 spectrophotometer (Hitachi, Ltd., Tokyo, Japan); circulating water multi-purpose vacuum pump (Gongyi Yuhua Instrument Co., Ltd., Zhengzhou, China); freeze dryer (Ningbo Scientz Biotechnology Co., Ltd., Ningbo, China); rotary evaporator (Shanghai xiande experimental instrument Co., Ltd., Shanghai, China); desk centrifuge 5418 (Eppendorf, Inc., Hamburg, Germany); BS–160A Automatic partial collector (Shanghai huxi analytical instrument, Shanghai, China); 1100 Series Gel size exclusion chromatograph (Agilent Technologies, Santa Clara, CA, USA); 1200 Infinitely high-performance liquid chromatography (Agilent Technologies, CA, USA); and TENSOR 27 near-infrared diffuse reflectance spectrometry (Germany BRUKER, Karlsruhe, Germany).

### 2.3. Experimental Process Chart

**Figure 1:**
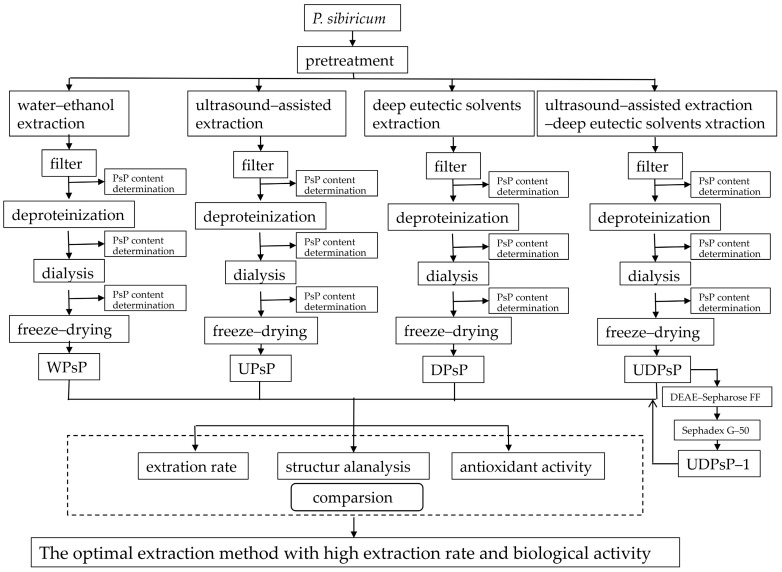
Experimental process chart. Abbreviations: WPsP, extracted PsP using WEE method; UPsP, extracted PsP using UAE method; DPsP, extracted PsP using DES method; UDPsP, extracted PsP using UAE–DES method; UDPsP–1, main component of separated and purified UDPsP.

Figure 1 shows the experimental process chart comparing different extraction methods.

### 2.4. Different Extraction Methods of PsP

#### 2.4.1. Pretreatment Process

After the *P. sibiricum* rhizome was cut into thin slices, the slices were dried in an oven at 45 °C to constant weight. The dried slices were then crushed, and the obtained powders were screened through an 80-micron mesh sieve. Finally, they were stored in a dryer.

#### 2.4.2. Water–Ethanol Extraction (WEE) Method of PsP

A total of 250 mg *P. sibiricum* powder was transferred to a round-bottom flask. Then, 150 mL of 80% ethanol was added to it, and the mixture solution was heated in a boiling water bath for 1 h using a Soxhlet extractor. After that, the hot mixture solution was filtered using 70 mm medium-speed filter paper, and the residue was washed with 80% hot ethanol three times. After the obtained residue and filter paper were transferred back to the flask, 150 mL of distilled water was added to it. The mixture solution was heated again in a boiling water bath for reflux in 1 h. Then, the filtrate was collected, protein was removed from the filtrate, and the remaining filtrate was dialyzed for removing small molecule substances after the hot mixture solution was filtered. Finally, the PsP powder was obtained through a freeze-drying process (freeze dryer, China), which was named water–ethanol extraction method–PsP (WPsP) [11].

#### 2.4.3. Ultrasound-Assisted Extraction (UAE) Method of PsP

The prepared *P. sibiricum* powder was weighed, and distilled water was added at a liquid-to-material ratio of 4:1 to form a 6.5 mL solution. Then, the PsP extraction was performed in a 50 mL centrifuge tube by using ultrasonic assistance at 82 W and 80 °C for 51 min. After cooling to room temperature, 26 mL of anhydrous ethanol was subsequently added into the centrifuge tube. And then, the centrifuge tube was shaken by using a Vortex mixing device (ORTEx Genius, Germany) for mixing samples. After that, the samples were placed in a refrigerator at 4 °C for 12 h. Then, the supernatant was discarded after the samples were centrifuged, and the sediment was further dissolved in 20 mL of distilled water. The processes of removing protein and dialysis were the same as in Section 2.4.2 after the PsP solution was filtered. Finally, the PsP powder was obtained and named ultrasound-assisted extraction method–PsP (UPsP) [13].

#### 2.4.4. Deep Eutectic Solvent Extraction (DES) Method of PsP

Deep eutectic solvents, which consisted of choline chloride (HBA) and 1,4–butanediol (HBD) with a molar ratio of 1:4, were used in this research, and the mixture was stirred using a magnetic stirrer (magnetic stirrers, Schwabach, Germany) to form the DES solution [18]. Then, the *P. sibiricum* powder was put into a beaker after it was weighed, and the DES solution was added subsequently into a beaker at a liquid-to-material ratio of 4:1. After that, the extraction was performed at 80 °C for 51 min. The subsequent operation steps were the same as in Section 2.4.3. Finally, the PsP powder was obtained and named deep eutectic solvent extraction method–PsP (DPsP).

#### 2.4.5. Ultrasound-Assisted Extraction–Deep Eutectic Solvent Extraction (UAE–DES) Method of PsP

The extraction method was established by our team in a previous study [19]. First, the DES was prepared, which was same as in Section 2.4.4. Then, the *P. sibiricum* powder was weighed before the DES solution was added at a liquid-to-material ratio of 4:1. After that, the extraction was performed using ultrasonic assistance at 82 W and 80 °C for 51 min. The subsequent operation steps were the same as in Section 2.4.3. Finally, the PsP powder was obtained and named the ultrasound-assisted extraction–deep eutectic solvent extraction method–PsP (UDPsP).

### 2.5. Separation and Purification of the PsP

PsP’s proteins were removed using the Sevage method, which was composed of chloroform and *n*–butanol at a ratio of 4:1. The PsP solution was mixed with Sevage reagent at a ratio of 4:1, and the mixture was shaken for 30 min. And then, the supernatant was collected. This operation was repeated until the sediment was completely removed. After that, the PsP solution was dialyzed for two days to remove small molecular substances using a 1000 Da membrane. Then, the concentrated PsP solution [by using a rotary evaporator at 45 °C (circulating water multi-purpose vacuum pump, China)] was freeze-dried into powder under −60 °C [19].

After the above operations were completed, the UDPsP was further purified by ion exchange chromatography and gel column chromatography. In this process, the DEAE–Sepharose Fast Flow (DEAE–Sepharose FF) was used for column packing in ion exchange chromatography. First, the UDPsP was dissolved in 2 mL of distilled water before the mixture was centrifuged at 10,000 rpm for 10 min. Then, the supernatant was slowly added to the column, and then, the DEAE–Sepharose FF was eluted in the following order: distilled water, 0.1 M, 0.2 M, 0.5 M, and 1 M NaCl, with 10 mL per tube. After that, the UDPsP solution at the peak position was collected (BS–160A Automatic partial collector, China) according to the elution curve.

After the operation of ion exchange chromatography was completed, the Sephadex G–50 column, which was fully swollen, was used to further purify UDPsP. The UDPsP, which passed through the ion exchange column, was dissolved in 2 mL of distilled water. After it was centrifuged at 10,000 rpm for 10 min, the supernatant was injected into the Sephadex G–50 column. Subsequently, elution was performed using distilled water. Then, the chromatogram was drawn according to the PsP concentration of each elution tube. After that, the PsP’s main component was obtained through freeze-drying, which was named separated and purified UDPsP (UDPsP–1).

### 2.6. Composition Analysis

#### 2.6.1. Measurement for PsP

The PsP samples were mixed with 0.2% anthrone–sulfuric acid solution at a 1:4 volume ratio. The absorbance value was measured at 582 nm (U–3900 spectrophotometer, Japan) [16]. The extraction yield and purity were calculated as shown Equation (1).

(1) $$R(\%)=\frac{C\times V\times N}{M}\times 100$$

Equation (1): R: extraction yield; C: PsP concentration; V: sample volume; N: dilution ratio; M: *P. sibiricum* mass.

#### 2.6.2. Measurement for Protein

The BCA protein assay kit was adopted to measure protein content in PsP solution. Firstly, a series of protein standard solutions with various concentrations were prepared. Then, 20 μL of each standard solution and PsP samples were added into different tubes. Subsequently, 200 μL of the BCA working solution was added to each tube. After they were incubated at 37 °C for 30 min, the absorbance value of each tube was measured at 562 nm [24].

### 2.7. Measurement for PsP’s Molecular Weight

The PsP’s molecular weight was measured by high-performance gel permeation chromatography (HPGPC). The PsP solution was filtered by a membrane, and then, it was analyzed using a TSK–gel G3000 PWXL column (maintained at 40 °C). The differential refractive index detector was employed by using a mobile phase of 0.05 M sodium sulfate. Then, a series of polyethyleneglycol (PEG) standards were used to establish a standard curve, so as to calculate the PsP’s molecular weights [25].

### 2.8. Measurement for PsP’s Monosaccharide Component

Firstly, the samples were hydrolyzed by adding 1 mL of distilled water and 1 mL of 4 M trifluoroacetic acid (TFA) before they were placed in an oven. Then, the samples were successively neutralized with NaOH and derivatized [26]. After that, these samples were incubated at 70 °C for 1 h under dark conditions. The mixture was extracted with chloroform after it was neutralized by using HCl. The supernatant was filtered with a 0.22 µm aqueous phase filter, and the filtrate was analyzed by high-performance liquid chromatography (HPLC) (1200 Infinitely high-performance liquid chromatography, USA) [27]. The detection conditions were the same as in previous research of our team [28].

### 2.9. Measurement for PsP’s Functional Groups

The PsP’s powder was mixed with KBr powder. The mixture was pressed into pellets after it was ground thoroughly in a mortar. After that, the Fourier transform infrared (FTIR) spectrometer was used to scan the pellet, so as to obtain the results of PsP’s functional groups.

### 2.10. In Vitro Antioxidant Activity

#### 2.10.1. DPPH and ABTS Radical Scavenging Rate

We adopted the method that was used by our team in previous research [19] to determine the DPPH and ABTS radical scavenging rate. The concentrations of the PsP solutions from different extraction methods were 0.5 mg·mL^−1^, 1 mg·mL^−1^, 2 mg·mL^−1^, 4 mg·mL^−1^, and 8 mg·mL^−1^. The concentrations of the purified UDPsP–1, which were calculated on the basis of equivalence, were 0.25 mg·mL^−1^, 0.5 mg·mL^−1^, 1 mg·mL^−1^, 2 mg·mL^−1^, and 4 mg·mL^−1^. The radical scavenging abilities were calculated as shown in Equation (2):

(2) $$\mathrm{Free}\;\mathrm{radical}\;\mathrm{scavenging}\;\mathrm{rate}\;(\%)=1-\frac{\mathrm{Aj}–\mathrm{Ai}}{\mathrm{Ao}}\times 100$$

Equation (2): Aj: absorbance of PsP sample; Ai: absorbance of control group; Ao: absorbance of blank group.

#### 2.10.2. Total Antioxidant Capacity

##### Phosphorus Molybdenum Complexation (PMC) Method

Firstly, 0.6 M H_2_SO_4_, 28 mM Na_3_PO_4_, and 4 mM H_8_MoN_2_O_4_ were mixed at a ratio of 1:1:1 to prepare phosphorus molybdenum complexation (PMC) solution. The PsP samples of different extraction methods and purified UDPsP–1 sample were mixed with the test solution; then, these mixtures were heated at 95 °C for 1.5 h [29]. Finally, the absorbance values of these mixtures were measured at 695 nm (SpectraMax I3X Enzyme marker, Torrance, CA, USA).

##### Ferric Reducing Antioxidant Power (FRAP) Method

The ferric reducing antioxidant power (FRAP) of PsP was determined by a FRAP assay kit. Firstly, a series of Fe_2_SO_4_ standard solutions with various concentrations were prepared. The PsP with different concentrations (0.5, 1, 2, 4, 8 mg·mL^−1^) of different extraction methods and the purified UDPsP–1 with different concentrations (0.25, 0.5, 1, 2, 4 mg·mL^−1^) were prepared. Then, 5 µL of PsP samples and 180 µL of working solution were added successively into each well of a 96-well plate. The reaction system was incubated at 37 °C for 5 min. The absorbance value was measured at 593 nm. The FRAP value of the PsP sample was calculated using the standard equation.

#### 2.10.3. In Vitro Catalase (CAT) Activity Measurement

The PsP with different concentrations (0.5, 1, 2, 4, 8 mg·mL^−1^) of different methods, and the purified UDPsP–1 with different concentrations (0.25, 0.5, 1, 2, 4 mg·mL^−1^) were prepared, respectively. Then, each PsP sample (10 µL) was added into each well of a 96-well quartz colorimetric plate. After that, catalase (CAT) solution (190 µL) was added to each well. The absorbance value (A1) was measured at 240 nm, and the operation was repeated after 1 min to obtain A2 [30]. The CAT activity (U·mL^−1^) was calculated as Equation (3).

(3) CAT(U/mL)=678×ΔA

Equation (3): CAT: catalase; ∆A: A1 − A2.

### 2.11. Statistical Analysis

A one-way analysis of variance (ANOVA) followed by Least Significant Difference (LSD) was utilized to compare the difference between different groups. A *p* < 0.05 was considered as a significant difference for all results. Each experiment was replicated three times, and all experimental data are shown as means ± SD. SPSS 27.0 (IBM, Armonk, NY, USA) was used to conduct the statistical analysis, and Origin 2022 (Origin Lab Inc., Hampton, MA, USA) and Microsoft Excel 2010 (Microsoft Corporation, Redmond, WA, USA) were used to draw statistical graphs.

## 3. Results

### 3.1. Comparisons of Extraction Yields of Different Extraction Methods for PsP

Table 1 shows the extraction yield of different methods for PsP, which indicates that the UDPsP’s extraction yield (45.08%) was the highest, followed by DPsP (34.88%), UPsP (20.53%), and WPsP (10.13%). The PsP contents of different methods at each extraction stage are shown in Table 1. The PsP’s pureness in each extraction method reached 98%, and the differences in pureness among them were not significant (*p* > 0.05), which ensured the comparability of the PsP’s in vitro antioxidant abilities of different extraction methods.

### 3.2. Separation and Purification of UDPsP 

**Figure 2:**
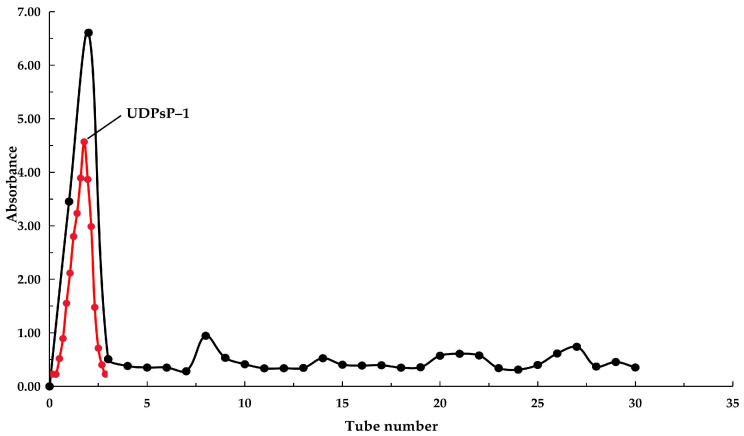
The elution curve of UDPsP passed through DEAE–Sepharose FF and Sephadex G–50 column. Note: black elution curve was UDPsP passed through DEAE–Sepharose FF; red elution curve was UDPsP passed through Sephadex G–50.

The UDPsP was used to further conduct separation and purification because its extraction yield was the highest. The elution curve of UDPsP passed through DEAE–Sepharose FF is shown in Figure 2, which showed that UDPsP (100 mg) was separated into two parts, including neutral PsP and acidic PsP. According to the peak area, it was known that neutral PsP was the main component of UDPsP. After the neutral PsP passed through the Sephadex G–50 column, the collected main PsP was named UDPsP–1 (Figure 2). The acidic PsP was composed of many small components, and it was difficult to further separate the acidic PsP, so we will research this in the future.

### 3.3. Comparison of Molecular Weight

**Figure 3:**
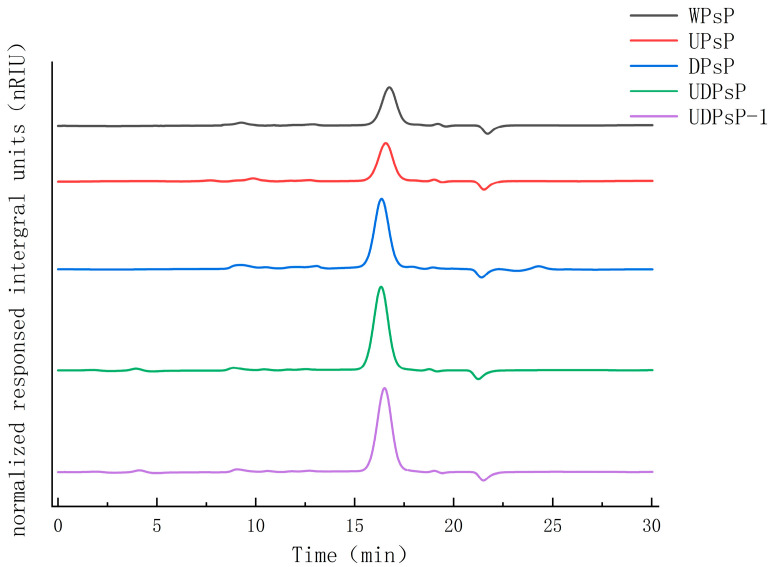
Comparison for molecular weight of purified UDPsP–1 and PsP from different methods.

The comparative results of PsP’s molecular weight are shown in Figure 3 and Table 2. The retention times of observed WPsP, UPsP, DPsP, UDPsP, and UDPsP–1 were, respectively, 16.83 min, 16.61 min, 16.40 min, 16.27 min, and 16.35 min, and the corresponding molecular weights were 2069 Da, 2118 Da, 2173Da, 2512 Da, and 2339 Da.

### 3.4. Composition for Monosaccharide Compositions

**Figure 4:**
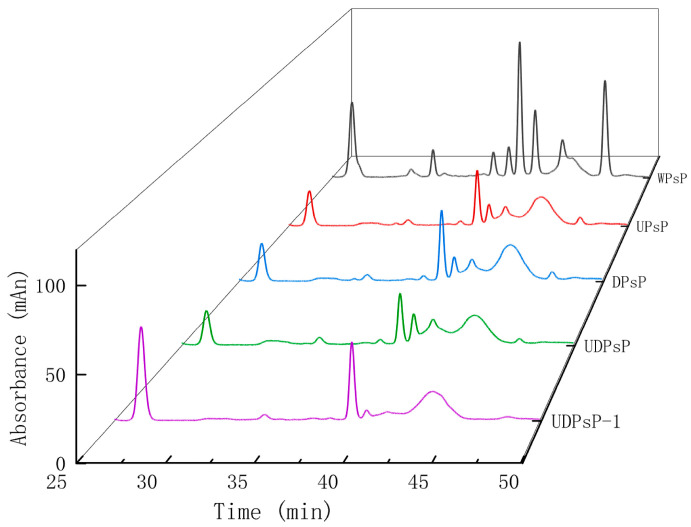
Comparison for monosaccharide composition of purified UDPsP–1 and PsP from different methods.

Figure 4 presents the results of monosaccharide compositions of purified UDPsP–1 and PsP from different methods. The results showed that the WPsP and DPsP comprised seven kinds of monosaccharides: mannose (Man), rhamnose (Rha), galactose acid (GalA), glucose (Glc), galactose (Gal), arabinose (Ara), and fucose (Fuc). The UPsP and UDPsP comprised six kinds of monosaccharides: Man, Rha, Glc, Gal, Ara, and Fuc. The monosaccharide kinds of UDPsP–1 had the least, only containing four kinds of monosaccharides: Man, Glc, Gal, and Ara.

Table 3 shows the result for molar ratios of monosaccharide composition of purified UDPsP–1 and PsP from different methods.

### 3.5. Comparison for Functional Groups

**Figure 5:**
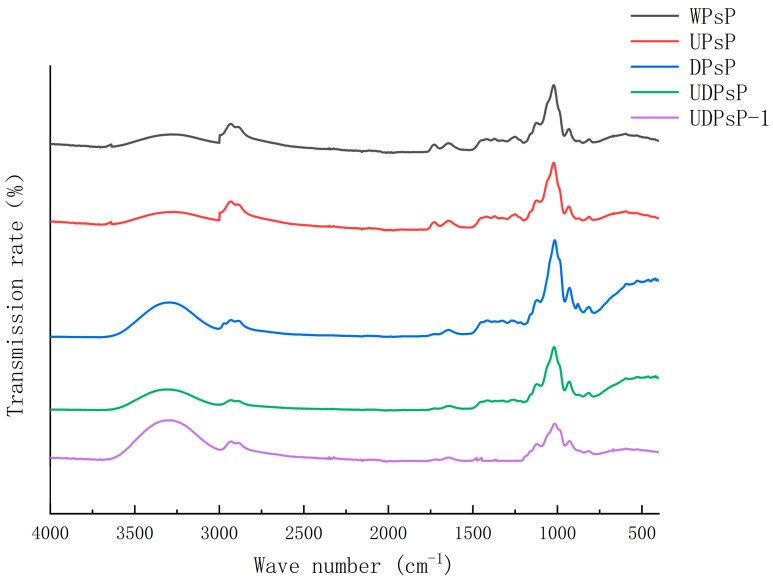
Comparison of infrared spectra of purified UDPsP–1 and PsP from different methods.

The results of infrared spectra of purified UDPsP–1 and PsP from different methods are shown in Figure 5. Their strong and wide peaks appear around 3250 cm^−1^, which indicates that all of them possess the O–H group. In addition, the five peaks occur, respectively, at 2900 cm^−1^, 1600 cm^−1^, 1400 cm^−1^, 1000 cm^−1^, and 950~700 cm^−1^, which indicates that they possess simultaneously C–H group, C=O group, methyl group, pyranose, and benzene ring skeleton. On the other hand, all of them show a special peak at 1200 cm^−1^ except for purified UDPsP–1, which indicates that there are no free carboxylic acids in the purified UDPsP–1.

### 3.6. Comparisons of Antioxidant Activity

#### 3.6.1. Comparison of DPPH and ABTS Radical Scavenging Ability

**Figure 6:**
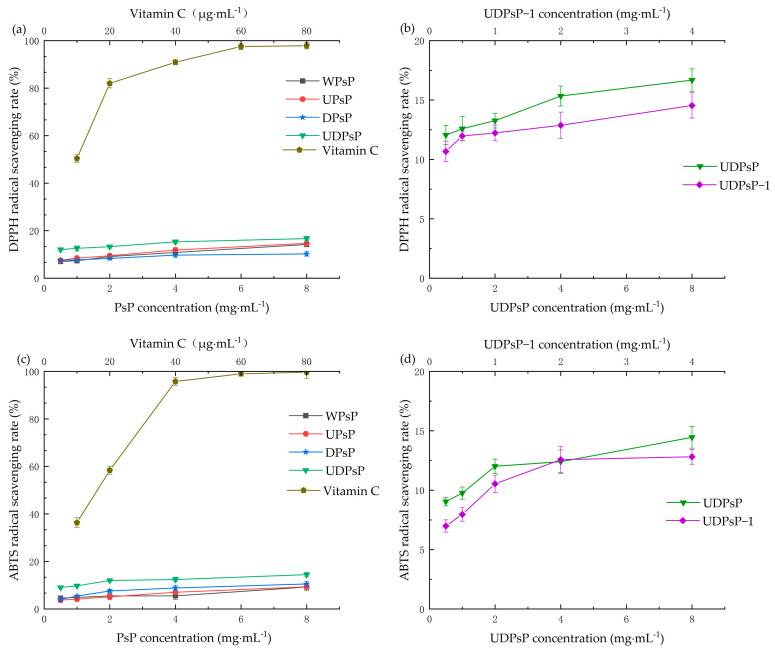
Comparisons of DPPH and ABTS radical scavenging rates. (**a**) Results of DPPH radical scavenging rates of WPsP, UPsP, DPsP, and UDPsP. (**b**) Results of DPPH radical scavenging rates of UDPsP and UDPsP–1. (**c**) Results of ABTS radical scavenging rates of WPsP, UPsP, DPsP, and UDPsP. (**d**) Results of ABTS radical scavenging rates of UDPsP and UDPsP–1.

The results indicated that the radical scavenging ability of WPsP, UPsP, DPsP, UDPsP, and UDPsP–1 increased gradually with the increase in their concentration. The differences in DPPH and ABTS radical scavenging abilities among WPsP, UPsP, DPsP, and UDPsP were significant (*F_DPPH_* = 6.936, *F_ABTS_* = 4.759, *p* < 0.05), and both DPPH and ABTS radical scavenging abilities of UDPsP were significantly higher than those of others (*t_DPPH(UDPsP_*_–_*_DPsP)_* = 2.794, *t_DPPH(UDPsP_*_–_*_UPsP)_* = 3.984, *t_DPPH(UDPsP_*_–_*_WPsP)_* = 4.336, *p* < 0.05; *t_ABTS(UDPsP_*_–_*_DPsP)_* = 5.141, *t_ABTS(UDPsP_*_–_*_UPsP)_* = 2.298, *t_ABTS(UDPsP_*_–_*_WPsP)_* = 2.704, *p* < 0.05). In addition, both DPPH and ABTS radical scavenging abilities of UDPsP were significantly higher than those of UDPsP–1(*t_DPPH_* = −21.585, *t_ABTS_* = −34.704, *p* < 0.05). Figure 6 shows the changed trend of DPPH and ABTS scavenging abilities of WPsP, UPsP, DPsP, UDPsP, and UDPsP–1. Table 4 shows the results of IC50 values of WPsP, UPsP, DPsP, UDPsP, and UDPsP–1, which indicated that UDPsP possessed the best DPPH and ABTS radical scavenging abilities.

#### 3.6.2. Comparison of Total Antioxidant Ability

**Figure 7:**
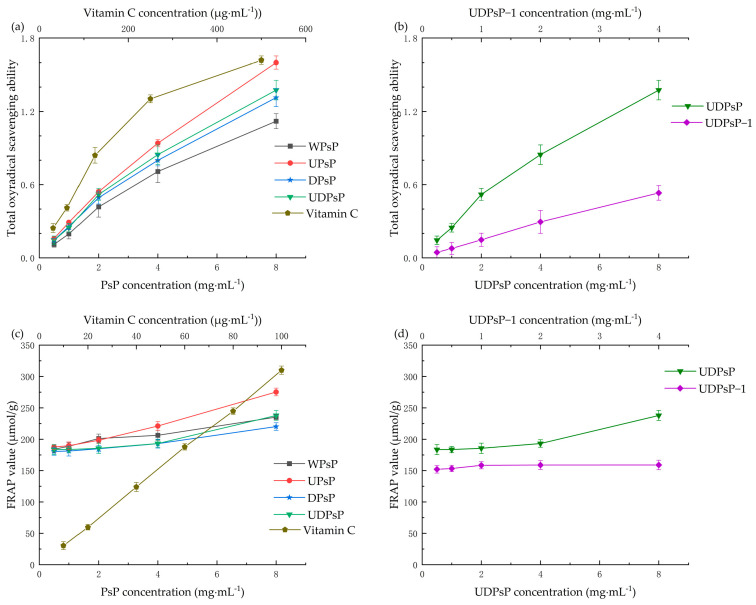
Comparisons of total antioxidant ability. (**a**) Results of the total antioxidant abilities of WPsP, UPsP, DPsP, and UDPsP using the PMC method. (**b**) Results of the total antioxidant abilities of UDPsP and UDPsP–1 using the PMC method. (**c**) Results of the total antioxidant abilities with FRAP of WPsP, UPsP, DPsP, and UDPsP. (**d**) Results of the total antioxidant abilities with FRAP of UDPsP and UDPsP–1.

Figure 7 shows the gradual increase in the total antioxidant abilities of WPsP, UPsP, DPsP, UDPsP, and UDPsP–1. There were no significant differences in the total antioxidant abilities among WPsP, UPsP, DPsP, and UDPsP (*F*_PMC_ = 0.814, *F_FRAP_* =1.067, *p* > 0.05). In addition, the total antioxidant ability of UDPsP was significantly higher than that of UDPsP–1 (*t*_PMC_ = 6.673, *t_FRAP_* = −21.176, *p* < 0.05).

#### 3.6.3. Comparison of In Vitro CAT Activity

**Figure 8:**
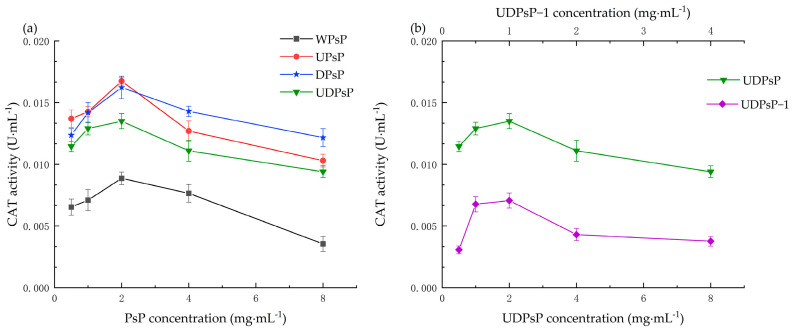
Comparisons of in vitro CAT activity. (**a**) Results of the in vitro CAT activity of WPsP, UPsP, DPsP, and UDPsP. (**b**) Results of the in vitro CAT activity of UDPsP and UDPsP–1.

The results showed that the improving effects of WPsP, UPsP, DPsP, and UDPsP increased gradually within the concentration range from 0.5 to 2 mg·mL^−1^ on CAT activity, and all of them decreased after 2 mg·mL^−1^. The improving effect of UDPsP–1 showed an enhancing trend within the concentration range between 0.25 and 1 mg·mL^−1^, and it began to decrease after the 1 mg·mL^−1^. There was no statistically significant difference in the improving effect among UPsP, DPsP, and UDPsP, and the improving effects of them were significantly higher than those of WPsP (*t_UDPsP–WPsP_* = 4.317, *t_DPsP–WPsP_* = 6.152, *t_UPsP–WPsP_* = 7.779, *p* < 0.05), respectively. In addition, the improving effect of UDPsP was significantly higher than that of UDPsP–1 (*t* = 6.344, *p* < 0.05). The above results are shown in Figure 8.

## 4. Discussion

At present, we have not found other teams that have adopted the UAE–DES method to extract PsP. Our team adopted the UAE–DES method to extract PsP under optimized extraction conditions, which is a new and comprehensive method in extracting PsP. This study focused on the comparison of the extraction and purification of PsP. In order to ensure comparability among different methods, we compared the WEE method, UAE method, and DES method with the UAE–DES method, because the UAE–DES method was proposed on the basis of the WEE method, UAE method, and DES method. In the future, we will further conduct more comparisons between the UAE–DES method and other methods, including microwave extraction, supercritical CO_2_ extraction, etc. Regarding whether separation and purification can affect the PsP’s performance, the UDPsP with the highest extraction yield was selected to conduct further separation and purification, and the UDPsP’s performances before and after separation and purification were further compared. The above comparisons mainly involved extraction yield, structural composition, and in vitro antioxidant activity. According to the results of the above three aspects, this study not only proved that the UAE–DES method could increase extraction yield and keep the structural composition and antioxidant activity of PsP to the greatest extent, but also confirmed that the extracted PsP should not be further separated and purified, which provided a technique for the efficient extraction and practical application of PsP. This can promote the utilization of PsP in the health product industry.

The extraction yield of UDPsP was higher than that of PsP extracted by the other three methods in our study, which proved that the UAE–DES method can integrate the advantages of the UAE method and DES method.

As we know, polysaccharide’s molecular weight relates to its structure, such as the polymerization’s degrees and the distribution of polysaccharide’s branch, which can lead to differences in the molecular weight [31]. In this study, the molecular weight of UDPsP (2512 Da) was the highest, and that of WPsP (2069 Da) was the lowest. The reason for this might be that different extraction methods need different extraction conditions, which can produce different influences on PsPs’ structural composition, and consequently affect molecular weight [32]. The WEE method requires higher temperatures and a longer amount of time in extraction, which can lead to partial degradation of the polysaccharides and result in smaller-molecular-weight fragments [33]. The UAE method can cause physical effects, such as cavitation, which might disrupt the polysaccharide structure and affect the molecular weight. The DES method can enhance solubility and preserve the integrity of polysaccharides, and can promote the extraction of substances with larger molecular weight [34], whose destructiveness might be smaller. The UAE–DES method can not only reduce the degradation effects during the extraction process, but also accelerate the interaction between the DESs and intracellular substances, which might preserve the structural composition of polysaccharide to the greatest extent and result in a higher molecular weight. The results in Section 3.3 proved the above features of different extraction methods. In addition, the molecular weight of UDPsP–1 was lower than that of UDPsP. The reason for this might be explained by the results in Section 3.4, which indicated that both kinds and amounts of UDPsP–1’s monosaccharides were less than those of UDPsP’s monosaccharides.

There were differences in the monosaccharide composition of PsP among the four methods and UDPsP–1. Compared to WPsP and DPsP, neither UPsP or UDPsP contained GalA. This result was different from the study of Yang et al. [35], whose results showed that the PsP extracted with the ethanol ultrasound extraction method contained GalA. The reason for this might be that our ultrasonic power (82 W) was higher than Yang et al.’s ultrasonic power (70 W). Ultrasound that generates high-frequency vibrations and liquid flow can implement strong mechanical shear force on polysaccharide molecules, which can disrupt the long-chain structure of GalA. With the increase in ultrasonic power, the destructiveness will worsen [36]. So, GalA was absent in both UDPsP and UPsP in our study, while it existed in the monosaccharide composition of Yang et al. Further, the PsP’s monosaccharide composition under the UAE–DES method was similar to that of the UAE method, and the PsP’s monosaccharide composition under the WEE method was similar to that of the DES method. Nonetheless, the UAE–DES method is still worth being developed, because its extraction yield is the highest, and UDPsP’s antioxidant ability is better than or equal to that of other PsPs, which also meant the missing GalA was not a crucial factor for PsP’s antioxidant ability. On the other hand, compared to UDPsP, UDPsP–1 did not contain Rha and Fuc, which might be because the process of separation and purification with ion exchange and gel filtration chromatography can cause further destructiveness for the monosaccharide composition of PsP [37].

The PsP’s infrared spectra of four different methods exhibited similar peak positions, all of which contained O–H group, C–H group, and C=O group. The results proved that UDPsP has similar polysaccharide properties to others, which explains why there was no significant difference in the PsP’s total antioxidant ability among four kinds of extraction methods in the results in Section 3.6.2. For UDPsP–1, its infrared spectrum was the same as UDPsP’s infrared spectrum, except for the carboxylic acids; therefore, it belongs to the neutral polysaccharide. The reason might be that carboxylic acids are characterized by acidity, which could be excluded in the separation process with anion exchange chromatography. The results can explain further why all antioxidant abilities of UDPsP–1 were lower than those of UDPsP in the results in Section 3.6.

Compared with the DPPH and ABTS scavenging abilities of PsP extracted with the other three methods and purified UDPsP–1, the UDPsP showed more advantages. The reason for this might be that the UAE–DES method can integrate the advantages of both the UAE method and DES method [11,38,39]. Therefore, the UDPsP’s solubility and the exposed amount of O–H and –COOH groups were not only higher than those of UPsP, but also higher than those of DPsP during the extraction process. Thus, the DPPH and ABTS scavenging rates of UDPsP were higher than those of other extraction methods. In addition, the UDPsP–1 contained less kinds of monosaccharide than UDPsP, and its acidic components were lost after separation and purification according to the results in Section 3.4 and Section 3.5, which led to a reduction in its antioxidant ability. This phenomenon can be proved by relevant biochemical mechanisms that acidic polysaccharides have negatively charged groups, such as GalA, which can cause them to have better biological activities, such as antioxidant ability [40,41,42]. Therefore, the DPPH and ABTS scavenging abilities of UDPsP were higher than those of UDPsP–1.

For the result that UDPsP did not show differences in total antioxidant ability with PsP extracted using the other three methods, this could be explained by the fact that the overall effect of functional groups with antioxidant ability was the same among different extraction methods. For the result that UDPsP was better than UDPsP–1 in total antioxidant ability, the reason might be that the purified UDPsP–1 lost some important functional groups with total antioxidant ability in the process of separation and purification [43].

UDPsP’s in vitro CAT activity was the same as UPsP and DPsP; the reason for this might be the same as the above explanation regarding total antioxidant ability. For the result that the CAT activity of UDPsP was higher than that of UDPsP–1, this might be attributed to UDPsP–1 belonging to neutral polysaccharide; therefore, it possessed a lower influence on CAT activity than the UDPsP. The above results also verified that the acidic environment was more conducive to improving the activity of CAT, which was consistent with the research results of others [44].

This study showed that UDPsP possessed more advantages in antioxidant ability than UDPsP–1. The result indicated that it is possible that there is a synergistically antioxidant effect between UDPsP–1 and the discarded part. Therefore, the PsP extracted using the UAE–DES method should not be separated and purified in the subsequent process, so as to keep the optimal antioxidant ability of UDPsP.

This study indicated that the UAE–DES method can not only improve the extraction yield, but also better maintain PsP’s antioxidant ability than PsP extracted using other methods, and the UDPsP should not be further separated and purified. In the next stage, we will explore the mechanism of UDPsP in improving PA-induced lipid ectopic deposition in L6 cells on the basis of this research and will study the comprehensive value of UDPsP in delaying sarcopenia obesity (SO). We hope these results can provide a comprehensive and integral theoretical basis for the utilization of *P. sibiricum* resources.

## 5. Conclusions

We concluded that UDPsP’s extraction yield and molecular weight were the highest, and it showed similar characteristics to PsP extracted using the other three methods; it also showed superior performance in DPPH and ABTS radical scavenging abilities. In addition, these advantages of UDPsP can also be found in the comparison with UDPsP–1. Therefore, combined with the consideration of the extraction yield, the UAE–DES method showed more advantages than the other extraction methods, and the UDPsP should not be further separated and purified. This study provided a better technique for the maximum utilization of *P. sibiricum* resources and improved PsP’s research field. Importantly, the findings suggest that UDPsP has strong potential as a natural dietary supplement. Its high extraction yield and strong antioxidant activity make it a valuable candidate for applications in the food, pharmaceutical, and nutraceutical industries. In the future, we will focus on the effects, mechanisms, and practical applications of UDPsP in relation to sarcopenic obesity, so as to further explore the value of UDPsP.

## Figures and Tables

**Table 1:** The PsP contents in different extraction methods at different stages.

Sample (mg)	Extraction Yield mg (%)	After Deproteinization mg (%)	Dialysis mg (%)	End Product mg (%)	Protein Residue %	Purity %
WPsP (700)	70.91 ± 5.18 (10.13 ± 0.74)%	45.43 ± 1.05 (6.49 ± 0.15)%	39.19 ± 2.66 (5.60 ± 0.38)%	23.80 ± 1.25 (3.40 ± 0.18)%	(0.63 ± 0.35)% *	(98.24 ± 0.47)% *
UPsP (700)	143.71 ± 5.18 (20.53 ± 1.43)%	95.44 ± 0.84 (13.63 ± 0.12)%	76.73 ± 1.26 (10.96 ± 0.18)%	57.24 ± 1.07 (8.18 ± 0.15)%	(0.57 ± 0.53)% *	(98.28 ± 0.30)% *
DPsP (700)	244.16 ± 4.76 (34.88 ± 0.68)%	158.38 ± 1.19 (22.63 ± 0.17)%	107.79 ± 0.42 (15.40 ± 0.06)%	67.42 ± 0.89 (9.63 ± 0.13)%	(0.33 ± 0.08)% *	(98.76 ± 0.17)% *
UDPsP (700)	315.56 ± 9.73 (45.08 ± 1.39)%	235.66 ± 1.02 (33.67 ± 0.86)%	154.64 ± 1.96 (22.09 ± 0.28)%	102.12 ± 0.94 (14.59 ± 0.13)%	(0.42 ± 0.31)%	(98.15 ± 0.32)%

Note: * comparison with UDPsP, *p* > 0.05.

**Table 2:** Result of molecular weight of purified UDPsP–1 and PsP from different methods.

Sample	Retention Times (min)	Mw (Da)
WPsP	16.83	2069
UPsP	16.61	2118
DPsP	16.40	2173
UDPsP	16.27	2512
UDPsP–1	16.35	2339

**Table 3:** Molar ratios of monosaccharide composition of purified UDPsP–1 and PsP from different methods.

Sample	Man	Rha	GlcA	GalA	Glc	Gal	Ara	Fuc
WPsP	1.97	0.34	0.00	2.21	1.00	1.96	1.29	2.58
UPsP	0.47	0.15	0.00	0.00	1.00	0.16	0.18	0.1
DPsP	0.39	0.12	0.00	0.17	1.00	0.16	0.29	0.08
UDPsP	0.49	0.08	0.00	0.00	1.00	0.28	0.48	0.07
UDPsP–1	0.86	0.00	0.00	0.00	1.00	0.05	0.09	0.00

Abbreviations: Man, mannose; Rha, rhamnose; GlcA, glucuronic acid; GalA, galactose acid; Glc, glucose; Gal, galactose; Ara, arabinose; Fuc, fucose.

**Table 4:** IC50 value of different extracts and vitamin C for DPPH and ABTS radical scavenging rate.

Sample	IC50 of DPPH Radical Scavenging Rate (mg·mL^−1^)	IC50 of ABTS Radical Scavenging Rate (mg·mL^−1^)
WPsP	60.83	45.51
UPsP	61.92	45.68
DPsP	57.15	62.36
UDPsP	54.77	41.64
UDPsP–1	28.46	38.29
Vitamin C	16.49	9.86

## Data Availability

The original contributions presented in this study are included in the article. Further inquiries can be directed to the corresponding author.

## Abbreviations

**Abbreviation**	**Full Name**
ABTS	2,20–azino–bis(3–ethylbenzothiazoline–6–sulfoniacid)
BSA	Bovine serum albumin
DESs	Deep eutectic solvents
DPPH	2,2–diphenyl–1–picrylhydrazyl
DPsP	Deep eutectic solvent extraction method–PsP
FRAP	Ferric reducing antioxidant power
*P. sibiricum*	*Polygonatum sibiricum*
PMC	Phosphorus molybdenum complexation
PsP	*Polygonatum sibiricum* Polysaccharide
TFA	Trifluoroacetic acid
UAE	Ultrasound-assisted extraction
UAE–DESs	Ultrasound-assisted extraction–deep eutectic solvents
UDPsP	Ultrasound-assisted extraction–deep eutectic solvent extraction method–PsP
UDPsP–1	Separated and purified UDPsP
UPsP	Ultrasound-assisted extraction method–PsP
WEE	Water–ethanol extraction
WPsP	Water–ethanol extraction method–PsP
